# Electroacupuncture ameliorates ulcerative colitis by suppressing ferroptosis via the JAK2/STAT3 signaling pathway

**DOI:** 10.1186/s13020-026-01337-9

**Published:** 2026-01-28

**Authors:** Tao Zhu, Hong-ye Wan, Zheng-yang Qu, Hong-kai Zhu, Lai-xi Ji, Jing Zhang

**Affiliations:** 1 School of Acu-Mox and Tuina, Chengdu University of Traditional Chinese Medicine, Chengdu, 611137 China; 2 Institute of Acupuncture and Moxibustion, China Academy of Chinese Medical Sciences, Beijing, 100700 China; 3 School of Acu-Mox and Tuina, Shanxi University of Chinese Medicine, Jinzhong, 030619 China; 4 Second Department of Acupuncture and Moxibustion, Affiliated Hospital of Shanxi University of Chinese Medicine, Taiyuan, 030032 China

**Keywords:** Electroacupuncture, Ulcerative colitis, Oxidative stress, Ferroptosis, Inflammation, JAK2/STAT3 signaling pathway, Intestinal barrier integrity

## Abstract

**Background:**

As an important component of external therapies in traditional Chinese medicine (TCM), the specific mechanism of acupuncture in improving UC has not been fully elucidated. This study investigates the regulatory effects of acupuncture on ferroptosis and the JAK2/STAT3 signaling pathway in colon epithelial cells of dextran sulfate sodium (DSS)-induced UC mice, thereby providing an in-depth exploration of the potential molecular mechanisms underlying acupuncture treatment for UC.

**Methods:**

In the first phase, using a sham electroacupuncture (SEA) group as a control, the effects of electroacupuncture (EA) on ferroptosis, intestinal mucosal barrier function, oxidative stress levels, and the inflammatory response in DSS-induced colon epithelial cells were investigated. Furthermore, the expression levels of the JAK2/STAT3 signaling pathway in colon tissue were examined. In the second phase, the ferroptosis-specific activator erastin was co-administered to further validate the critical mechanistic role of ferroptosis inhibition in EA treatment. In the third phase, the JAK2-specific inhibitor AG490 was used to intervene in UC. A comparative analysis was conducted to assess the effect equivalence between JAK2/STAT3 pathway inhibition and EA treatment for UC, further clarifying the JAK2/STAT3 pathway as a key regulatory target of acupuncture in UC treatment.

**Results:**

Compared to the control (Con) group, the DSS group showed significant upregulation of ferroptosis-related indicators, impaired intestinal mucosal barrier function, markedly increased levels of oxidative stress and inflammatory response, along with upregulated expression of the JAK2/STAT3 signaling pathway. Compared to the DSS group, the DSS + EA group exhibited significant improvement in colon histopathological damage, a substantial reduction in ferroptosis levels in colon epithelial cells, and corresponding downregulation of JAK2 and STAT3 expression levels. Notably, the therapeutic effects of the DSS + EA group were superior to those of the DSS + SEA group. The ferroptosis-specific activator erastin reversed the anti-ferroptosis effects of EA and its protective effects on the colon. In addition, the effect of EA treatment in ameliorating ferroptosis and colon injury was comparable to the intervention with the JAK2-specific inhibitor AG490.

**Conclusions:**

EA may alleviate ferroptosis in colonic epithelial cells by inhibiting the JAK2/STAT3 pathway, significantly reducing oxidative stress injury, improving intestinal mucosal barrier integrity, and inhibiting the DSS-induced inflammatory cascade in UC mice. This study provides important modern scientific evidence for the application of acupuncture therapy in treating gastrointestinal diseases.

**Supplementary Information:**

The online version contains supplementary material available at 10.1186/s13020-026-01337-9.

## Introduction

Ulcerative colitis (UC) is a chronic non-specific inflammatory bowel disease, pathologically characterized primarily by focal ulcer formation and persistent chronic inflammation in the colonic mucosa [1]. Epidemiological reports indicate that the incidence of UC is increasing annually, most commonly affecting individuals aged 20–40 years [2]. The disease not only imposes a significant impact on patients’ physical and mental health but also places a substantial economic burden on both families and the societal healthcare system [3]. Existing evidence suggests that the pathogenesis of UC involves significant multifactorial complexity, encompassing genetic susceptibility, immune dysregulation, imbalances in the gut microbiota, and other pathological factors [4, 5]. Among these, the persistent inflammatory response in the colonic mucosa is recognized as a key pathological driver of disease initiation and progression [6]. During the pathological course of UC, sustained inflammation directly disrupts the integrity of tight junction structures in intestinal epithelial cells, allowing pathogenic microorganisms to penetrate the compromised intestinal mucosal barrier [7]. This, in turn, activates the immune system and triggers a cascading amplification of the inflammatory response [8]. Consequently, mitigating intestinal inflammation and repairing the damaged mucosal barrier have emerged as critical therapeutic targets in UC management. However, the specific mechanisms underlying acupuncture’s regulation of the intestinal inflammatory response and its protective effects on the mucosal barrier in UC remain incompletely understood [9, 10].

It is noteworthy that the intestinal mucosal barrier injury, as a key initiating factor in the pathogenesis of UC [11], can trigger a series of pathophysiological alterations, including disruption of redox homeostasis [12], oxidative stress cascades [13], and peroxidation of biomacromolecules [14]. These aberrant responses act synergistically to drive and sustain the persistent pathological state of UC. Although acupuncture has shown potential therapeutic value, the specific downstream molecular mechanisms through which it exerts its therapeutic effects, such as antagonizing oxidative stress and promoting mucosal repair, remain to be fully elucidated [15, 16]. Consequently, the elucidation of the key molecular mechanisms of acupuncture in mitigating UC is crucial for advancing scientific understanding.

It is well-established that iron is an essential element for ferroptosis [17]. Excess ionic iron leads to “iron accumulation,” subsequently resulting in an iron-dependent mode of cell death known as ferroptosis [18, 19]. Growing evidence demonstrates that inhibiting ferroptosis can effectively ameliorate intestinal pathology in UC mice [19, 20]. Specifically, during the development and progression of UC, ferroptosis exacerbates the inflammatory cascade and disrupts the integrity of the intestinal mucosal barrier by mediating lipid peroxidation damage in intestinal epithelial cells, thereby accelerating disease progression [21, 22]. Existing research reveals a complex regulatory network linking ferroptosis, oxidative stress, and inflammatory responses [23, 24]. This distinct mechanism establishes ferroptosis as a key potential target for therapeutic intervention in UC. Although some studies have confirmed that acupuncture can modulate ferroptosis [16], the specific molecular mechanisms by which acupuncture regulates the ferroptosis pathway in UC have not yet been systematically investigated.

The JAK2/STAT3 signaling pathway, as a highly conserved evolutionarily related signal transduction pathway, plays a critical role in regulating biological processes such as cell proliferation, differentiation, apoptosis, and inflammatory responses [25–27]. In the pathological progression of UC, abnormal activation of the JAK2/STAT3 pathway promotes disease advancement by exacerbating the inflammatory cascade, disrupting the integrity of the intestinal mucosal barrier, and inducing apoptosis in intestinal epithelial cells [28–30]. Notably, the JAK2/STAT3 pathway is also involved in the regulatory network of ferroptosis; however, its specific mechanism of action exhibits significant disease-specificity and is closely associated with underlying pathophysiological features [31].

This study investigates the potential therapeutic role of acupuncture in UC, aiming to demonstrate that acupuncture exerts its effects through multi-target mechanisms, specifically by ameliorating intestinal mucosal barrier damage, counteracting oxidative stress, suppressing inflammatory responses, and alleviating ferroptosis. In particular, we elucidate that the protective effects of acupuncture are primarily mediated through the regulation of the JAK2/STAT3 signaling pathway. These findings suggest that acupuncture, as a promising external therapy, exhibits significant clinical potential in the treatment of UC, while also providing an innovative theoretical foundation and novel therapeutic strategies for UC management.

## Materials and methods

### Animals and ethics

Male C57BL/6 mice (6–8 weeks old, weighing 18 ± 2 g) under specific pathogen-free (SPF) conditions were purchased from Vital River Laboratory Animal Technology Co., Ltd. (Beijing, China; Animal Production License No.: SCXK(Jing) 2022-0006). The mice were reared in the experimental animal facility of Shanxi University of Chinese Medicine, under controlled conditions with a temperature of 23 ± 2 °C, relative humidity of 40–60%, and a 12-h light/dark cycle. All mice underwent a 1-week acclimatization period prior to the formal experiments. All animal experimental procedures were strictly conducted in accordance with protocols approved by the Animal Ethics Committee of Shanxi University of Chinese Medicine (Ethics Approval No.: 2021DW256) and conformed to *the Guide for the Care and Use of Laboratory Animals* by the National Institutes of Health (1996).

### Experimental design

In the first phase, 40 mice were randomly assigned into four groups (n = 10 per group): the control group (Con), the DSS group (DSS), the DSS plus EA group (DSS + EA), and the DSS plus sham EA group (DSS + SEA).

In the second phase, 40 mice were randomly divided into four groups (n = 10 per group): the Con, the DSS, the DSS + EA, and the DSS plus EA and Erastin group (DSS + EA + Erastin).

In the third phase, 50 mice were randomly allocated into five groups (n = 10 per group): the Con, the DSS, the DSS + EA, the DSS plus Tyrphostin AG490 group (DSS + AG490), and the DSS plus vehicle group (DSS + vehicle).

### Animal models

**Fig. 1:**
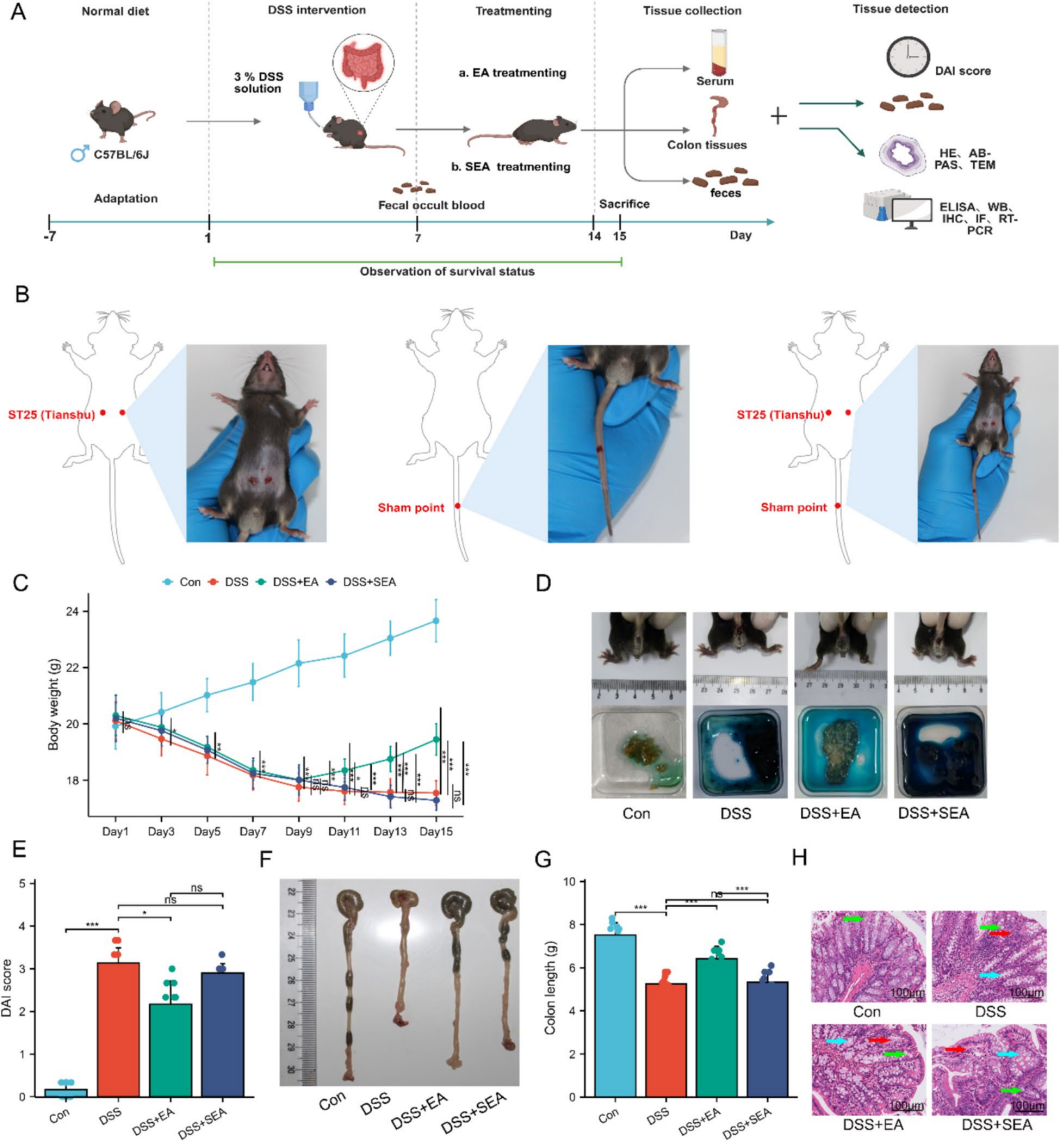
EA alleviates symptoms in UC mice. **A** Experiments protocols. **B** Location of ST25 (Tianshu) acupoint. **C** Body weight (n = 10). **D** Appearance of the mouse anus and fecal occult blood test. **E** DAI score (n = 10). **F** Macroscopic view of colons. **G** Colon length (n = 10). **H** HE staining of colonic tissues. Scale bars: 100 μm. Green arrows indicate glandular structures; blue arrows denote crypt structures; red arrows highlight inflammatory cell infiltration. Data are expressed as mean ± SD. ^*ns*^*P* > 0.05, *P < 0.05, **P < 0.01, ***P < 0.001

Based on established literature [32], the UC mouse model was induced in this study by providing free access to drinking water containing 3% dextran sulfate sodium (DSS, MP Biomedicals, USA) for 7 days (Fig. 1A). Mice in the Con group received sterile drinking water without DSS. Successful model establishment was confirmed by the presence of diarrhea, weight loss, soft stool consistency, and a positive fecal occult blood test [33].

### Intervention methods

1. Following the 7-day modeling period, mice in the EA group received electroacupuncture intervention bilaterally at the “Tianshu” (ST25) acupoint (Fig. 1B).

Acupoint localization was performed with reference to the common animal acupoints and atlases in Experimental Acupuncturology [34], the location of ST25 at a point 7/12 from the xiphoid process and 5/12 from the pubic symphysis along the ventral midline, 3 mm lateral to the midline. Mice were anesthetized with 2% isoflurane (0.5 L/min) for induction, followed by maintenance with 1% isoflurane (0.5 L/min). The acupoint areas were fully exposed and locally shaved. Sterile acupuncture needles (0.18 mm × 13 mm; Huatuo, China) were inserted vertically to a depth of 2–3 mm subcutaneously. The needles were then connected to the HANS100A type Han's acupoint stimulator (Nanjing Jisheng Medical Technology, China), delivering bilateral synchronous stimulation at parameters of 10 Hz frequency and 1 mA intensity for 20 min. This intervention was administered once daily for 7 consecutive days. Throughout the treatment period, the general behavior and activity of the mice, as well as the local response at the needling sites, were closely monitored to ensure the procedure did not induce excessive stress in the animals.

(2) The procedure for the SEA group was identical to that of the EA group, except that the EA stimulation was applied to a non-acupoint located on the tail of the mice (serving as a control, located at the midline of the tail, see Fig. 1) [35, 36]. The acupuncture needle was inserted to a depth of 2–3 mm, and an auxiliary needle was placed approximately 3 mm from this point, inserted to a depth of 1–2 mm. Subsequently, both the acupuncture needle and the auxiliary needle were connected to the electroacupuncture device.
(3) Mice in the Con and DSS groups received only the anesthesia regimen (identical to that used for the EA group) without any additional interventions.
(4) In the DSS + EA + Erastin group, mice received an intraperitoneal injection of the ferroptosis inducer erastin (HY-15763, 40 mg/kg; MedChemExpress, USA) one hour prior to the daily acupuncture session. The erastin solution was prepared in a vehicle consisting of 15% Cremophor EL-40 (HY-Y1890, MedChemExpress, USA) and 85% saline (Sichuan Kelun Pharmaceutical, China). It was administered intraperitoneally at a dosage of 40 mg/kg per day, adjusted according to the individual body weight of each mouse [37].
(5) Mice in DSS + AG490 group received intraperitoneal injections of the JAK2-specific inhibitor AG490 (5 mg, MCE, USA). The AG490 solution was prepared in a vehicle composed of 2% dimethyl sulfoxide (DMSO), 40% polyethylene glycol 300 (PEG 300, Selleck, USA), and 58% saline. It was administered intraperitoneally at a dosage of 5 mg/kg [38], based on the mice’s body weight. In the DSS + vehicle group, mice received daily injections of an equivalent volume of the saline vehicle solution.

### General condition monitoring

From the start of the experiment, body weight changes and survival status of the mice in each group were measured and assessed every 2 days at 8:00 AM.

### Disease activity index (DAI) assessment and colon length measurement

On day 15 of the experiment, changes in body weight, stool consistency, and the degree of fecal bleeding in the mice were observed and quantitatively scored according to the DAI criteria [39]. The DAI was calculated using the formula: DAI = (Weight Loss Score + Stool Consistency Score + Fecal Blood Score)/3. The percentage weight loss was calculated as: (Last-intervention weight—Initial weight)/Initial weight × 100%. Detailed DAI scoring criteria are provided in Supplementary Table 1.

After the intervention period, the mice were dissected, and the entire length of the colon from the cecum to the rectum was measured.

### H&E staining and AB-PAS staining

Mouse colon tissues were fixed in 4% neutral buffered paraformaldehyde solution and subsequently processed into paraffin-embedded blocks. The tissue blocks were trimmed and serially sectioned with a fully automated microtome to obtain 5-μm thick sections. The sections were deparaffinized in xylene and rehydrated through a graded ethanol series (100%, 95%, 80%, 70%). Histochemical staining was performed with the conventional H&E method and the Alcian Blue-Periodic Acid Schiff (AB-PAS) staining technique. After staining, the sections were dehydrated through a graded ethanol series, cleared, and mounted with neutral balsam. Colonic lesions in each group were observed under an optical microscope and evaluated according to the Geboes histological scoring criteria [40]. In AB-PAS staining, blue-stained areas represent acidic mucin substances, while red-stained areas correspond to glycogen and neutral mucin substances.

### Transmission electron microscopy (TEM) detection

Fresh colon tissues (1 mm^3^) from each group were selected and placed in EP tubes containing 2.5% glutaraldehyde fixative. After rinsing with 0.1% phosphate buffer, the tissues were re-fixed with 1% osmium acid, followed by thorough washing with PBS buffer. Dehydration was performed using a graded acetone series, and routine embedding was carried out with epoxy resin 812. The embedded samples were placed in a 37 °C incubator overnight, then sequentially transferred to 45 °C and 60 °C incubators for polymerization. Ultrathin sections were prepared, double-stained with uranyl acetate and lead citrate, and the ultrastructural morphology of mitochondria in the colon tissues was observed under a transmission electron microscope.

### Inflammatory factor and oxidative stress indicator assays

Serum samples were collected to measure the levels of inflammatory factors, including interleukin-1β (IL-1β, E-EL-M0037, Elabscience, China), interleukin-6 (IL-6, E-EL-M0044, Elabscience, China), and tumor necrosis factor-α (TNF-α, E-EL-M3063, Elabscience, China), as well as to assess oxidative stress-related indicators encompassing glutathione (GSH, E-BC-K030-M, Elabscience, China), superoxide dismutase (SOD, Elabscience, E-BC-K020-M, China), and malondialdehyde (MDA, E-EL-0060, Elabscience, China). All measurements were performed using ELISA kits according to the manufacturers’ instructions.

### Colon Fe^2^⁺ detection

Colon tissue samples from mice in each group were collected to determine Fe^2^⁺ levels. The Fe^2^⁺ content was detected with a colorimetric method, strictly following the instructions of the commercial kit (Jiangsu Addison Biotechnology, China). The optical density was measured at 593 nm with a microplate reader, and the iron ion concentration in the samples was calculated based on the standard curve.

### Immunohistochemistry (IHC)

Colon tissues were fixed in 4% paraformaldehyde and processed into paraffin-embedded blocks. The tissue blocks were then continuously sectioned to obtain 5 μm thick sections. These sections underwent gradual dewaxing followed by rehydration through a series of alcohol solutions, culminating in a final rinse with pure water. Antigen retrieval was performed using sodium citrate buffer (pH 6.0) under microwave heating at 98 °C. After natural cooling to room temperature, the tissue sections were incubated in 3% hydrogen peroxide solution for 10 min to block endogenous peroxidase activity, followed by blocking with 3% bovine serum albumin (BSA) for 50 min. Diluted primary antibodies (including anti-ZO-1, anti-Occludin, anti-Claudin 1, anti-TFR1, anti-GPX4, and anti-ACSL4) were applied, and the sections were incubated at 4 °C overnight. The next day, secondary antibody incubation was performed at room temperature for 50 min. Color development was achieved using a DAB solution, followed by counterstaining with hematoxylin. The sections were then dehydrated and mounted. Images were captured with an optical microscope (BX51; Olympus, Tokyo, Japan). Four random fields of view were selected for positive cell counting, and the mean optical density value, representing protein expression levels, was calculated using ImagePro Plus 6.0 software. The concentrations of the antibodies used are detailed in Supplementary Table 2.

### Immunofluorescence (IF)

Paraffin-embedded colon tissue sections were sequentially subjected to deparaffinization and rehydration, antigen retrieval, and blocking with 5% BSA. The sections were then incubated with a primary antibody against ROS (1:200 dilution) at 4 °C overnight. The following day, after thorough washing with PBS, the sections were incubated in the dark at room temperature with fluorescent secondary antibodies Goat Anti-Mouse IgG H&L (Alexa Fluor^®^ 555), followed by counterstaining with DAPI (Solarbio, China). The sections were mounted with an anti-fade mounting medium (Beyotime, P0131, China). Images were acquired using a fluorescence microscope (Nikon, Japan). Three random fields of view were selected per sample, and image analysis was performed using CaseViewer 2.4 image analysis software (3D Histech, Hungary). Quantitative analysis of ROS-positive cells was conducted using ImagePro Plus 6.0 software to determine protein expression levels, with the average value from the three fields representing the final measurement for each sample. The concentrations of the antibodies used are detailed in Supplementary Table 2.

### Western blotting (WB)

Approximately 100 mg of colon tissue from mice in each group was homogenized and lysed to obtain the protein supernatant. The protein concentration was determined using a BCA protein assay kit to calculate the loading amount. Proteins were separated by SDS-PAGE electrophoresis and transferred onto a PVDF membrane. The membrane was then blocked with 5% skimmed milk at 37 °C for 1 h. Subsequently, it was incubated with primary antibodies (anti-JAK2, anti-STAT3, anti-p-JAK2, anti- p-STAT3, anti-β-actin) at 4 °C overnight, followed by incubation with a secondary antibody at room temperature for 2 h the next day. After washing with T-BST, the membrane was scanned, and the images were captured using a chemiluminescence/fluorescence image analysis system (5200Multi, Tanon, China). The results were analyzed with ImageJ software, and the relative expression level of the target protein was determined by calculating the ratio of the gray value of the target protein band to the corresponding gray value of the internal reference protein band. Antibody concentration details are provided in Supplementary Table 3.

### Real-time quantitative polymerase chain reaction (RT-qPCR)

Total RNA was extracted from colon tissues using a Trizol reagent kit, and its concentration and purity were measured by UV spectrophotometry. The RNA was reverse-transcribed into cDNA using a reverse transcription kit. Using the total mRNA as a template, cDNA was synthesized for PCR amplification detection. The reaction mixture consisted of: 1 µL qPCR primer, 1 µL cDNA product, and 10 µL SYBR Green qPCR Master Mix (2 ×). The amplification conditions were pre-denaturation at 95 °C for 30 s, followed by 40 cycles of 95 °C for 15 s and 60 °C for 30 s (annealing/extension). GAPDH was used as the internal reference gene, and the relative expression levels of JAK2 and STAT3 mRNA were calculated using the 2^−ΔΔCt^ method. Detailed primer sequences are listed in Supplementary Table 4.

### Statistical analysis

Statistical analysis was performed using GraphPad Prism 8.0 software (GraphPad Software, Inc., San Diego, CA, USA). Measurement data conforming to a normal distribution are expressed as the mean ± standard deviation (SD). Comparisons among multiple groups were conducted using one-way analysis of variance (ANOVA). For multiple comparisons between groups, the LSD test was applied when variances were homogeneous, and the Tamhane test was used when variances were heterogeneous. *P*-value of less than 0.05 (P < 0.05) was considered statistically significant. Statistical significance is defined as follows: ^*ns*^*P* > 0.05, *P < 0.05, **P < 0.01, ***P < 0.001.

## Results

### EA alleviates symptoms in UC mice

To establish the UC mouse model, the animals were administered 3% DSS solution freely in drinking water for 7 consecutive days (Fig. 1C, D). Results indicated that after 1 week of DSS intervention, the mice exhibited typical clinical symptoms, including significant weight loss, perianal soiling, and loose stools, accompanied by a strong positive fecal occult blood test. These collective changes confirmed the successful establishment of the UC mouse model.

Based on observations in DSS-induced UC mice, this study further investigated the therapeutic effects of EA stimulation at ST25. To clarify the specific therapeutic effects of needling ST25, a control group receiving EA at a SEA was included. Consistent with previous findings [41], EA stimulation at ST25 significantly ameliorated DSS-induced UC symptoms. Specifically, EA at ST25 markedly reduced the DAI in DSS-treated mice, primarily manifested as attenuated weight loss, reduced rectal bleeding, improved stool consistency, and increased colon length (Fig. 1E–G). Histopathological analysis revealed significant damage to the intestinal mucosal layer, destruction of crypt architecture, and inflammatory cell infiltration in DSS-induced UC mice. Compared to the DSS group, these pathological alterations were significantly improved in the DSS + EA group, whereas no marked improvement was observed in the DSS + SEA group (Fig. 1H). Notably, the therapeutic effects of EA at ST25 were more pronounced than those of SEA. In summary, EA at ST25 effectively alleviated UC symptoms.

### EA ameliorates intestinal mucosal barrier damage in UC mice

**Fig. 2:**
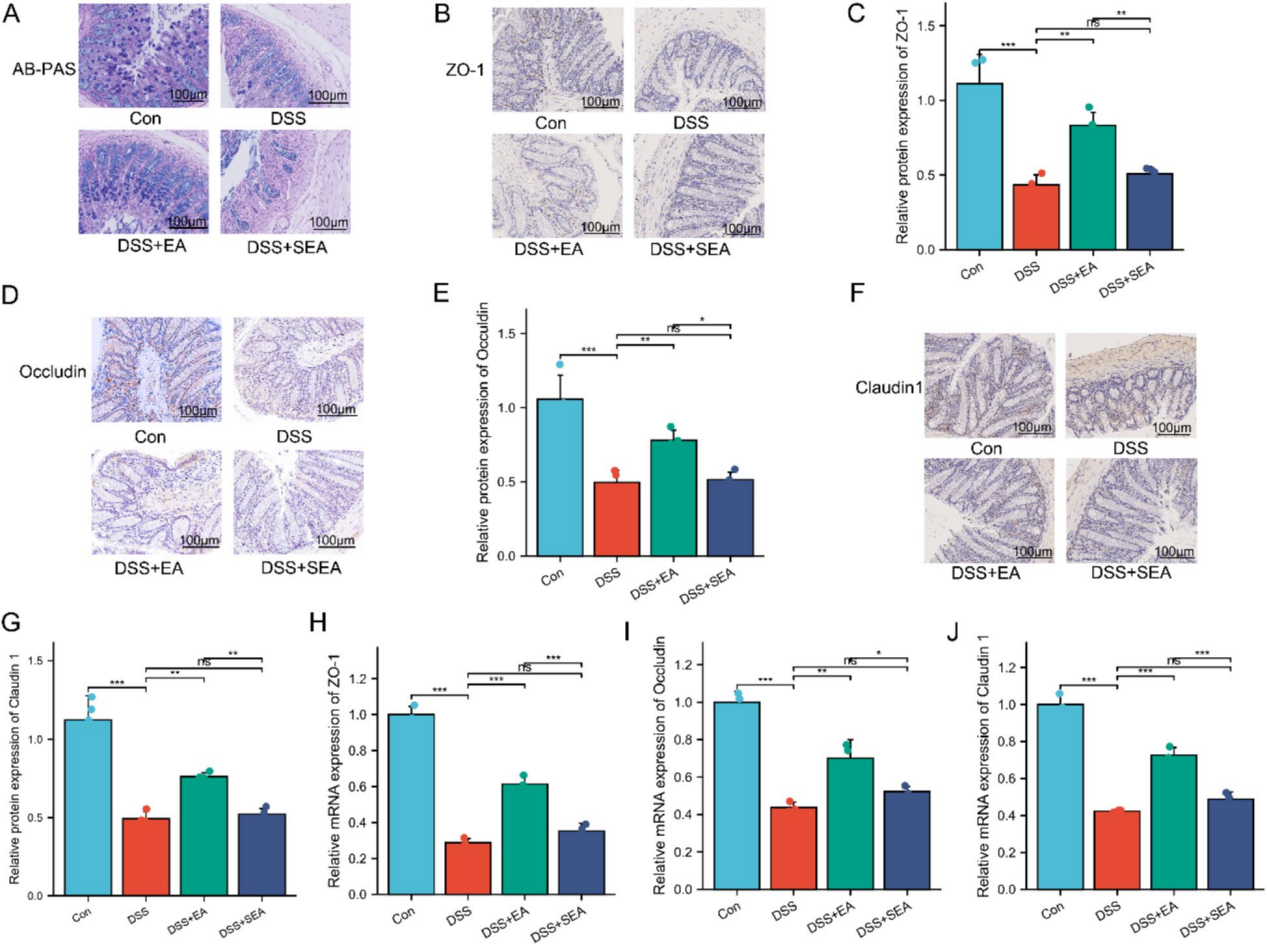
EA ameliorates intestinal mucosal barrier damage in UC mice. **A** AB-PAS staining of colonic tissues. Scale bars: 100 μm. **B**–**G** Immunohistochemical analysis of ZO-1, Occludin, and Claudin 1 in colonic tissues (n = 4). Scale bars: 100 μm. **H**–**J** mRNA levels of ZO-1, Occludin, and Claudin 1 in colonic tissues (n = 3). Data are expressed as mean ± SD. ^*ns*^*P* > 0.05, *P < 0.05, **P < 0.01, ***P < 0.001

Mucin secreted by goblet cells serves as a key effector molecule of intestinal barrier function, and its expression can be evaluated by AB-PAS staining [42]. AB-PAS staining revealed that, compared to the Con group, the DSS group exhibited a reduction in the number of colonic goblet cells and decreased mucin secretion. However, following EA treatment, the number of colonic goblet cells was significantly increased, and the mucin secretion function was markedly enhanced (Fig. 2A). In summary, these pathological changes were significantly improved after EA treatment, and EA demonstrated superior therapeutic efficacy compared to SEA.

In addition, we further evaluated the protective effects of EA on the intestinal mucosal barrier in UC mice. Immunohistochemistry results showed that the expression levels of the tight junction proteins ZO-1, Occludin, and Claudin-1 in the DSS + EA group were higher than those in the DSS group and the DSS + SEA group (Fig. 2B–G). RT-qPCR analysis further confirmed that the mRNA expression of ZO-1, Occludin, and Claudin-1 showed a similar trend (Fig. 2H–J). These findings suggest that EA can effectively improve intestinal epithelial barrier integrity and maintain intestinal homeostasis.

### EA alleviates colonic inflammation and oxidative stress in UC mice

**Fig. 3:**
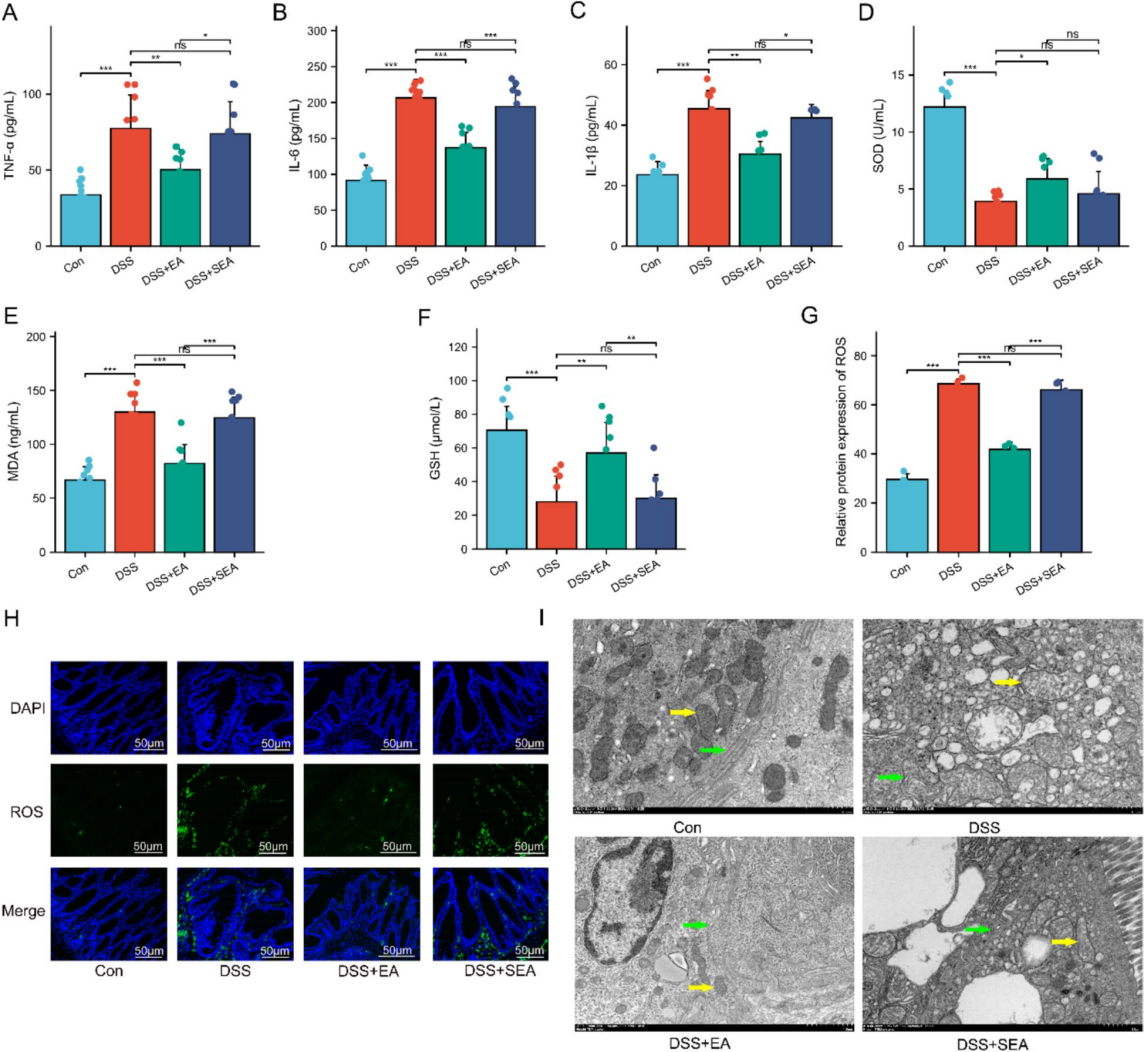
EA alleviates colonic inflammation and oxidative stress in UC mice. **A**–**C** Serum levels of TNF-α, IL-6, and IL-1β (n = 10). **D**–**F** Levels of SOD, MDA, and GSH in the colon (n = 10). **G**, **H** Immunofluorescence analysis of ROS (green) in colonic tissues (n = 4). Cell nuclei were stained with DAPI (blue). Scale bars: 50 μm. **I** The mitochondrial structure of the colon tissue was observed by TEM. Mitochondria, as indicated by yellow arrows; Rough endoplasmic reticulum, as indicated by purple arrows. Scale bars = 1 µm. Data are expressed as mean ± SD. ^*ns*^*P* > 0.05, *P < 0.05, **P < 0.01, ***P < 0.001

During the pathogenesis and progression of UC, damage to the intestinal mucosal barrier triggers an inflammatory response, which induces a cascade of immune regulatory responses and the release of various pro-inflammatory cytokines [43]. Results showed that the levels of TNF-α, IL-6, and IL-1β were significantly elevated in the DSS group compared to the Con group. After EA treatment, the serum levels of TNF-α, IL-6, and IL-1β were significantly reduced compared to those in the DSS group and the DSS + SEA group (Fig. 3A–C). These findings suggest that EA treatment exerts significant anti-inflammatory effects.

Oxidative stress is an important pathological mechanism of UC [44]. We assessed the expression levels of oxidative stress-related markers (SOD, MDA, GSH, ROS). The results indicated that DSS-induced UC mice exhibited a marked state of oxidative stress imbalance, characterized by significantly reduced serum SOD and GSH activities, decreased colon ROS levels, and a significant increase in MDA content. After EA treatment, the mice demonstrated a stronger resistance to DSS-induced oxidative stress, manifested as significantly increased serum SOD and GSH levels, elevated colon ROS expression, and significantly decreased MDA expression (Fig. 3D–H). It is noteworthy that the trends in these antioxidant indicators were consistent with the expression levels of the aforementioned inflammatory factors. Elevated ROS levels can exacerbate mitochondrial damage, primarily manifested as irreversible ultrastructural pathological changes such as mitochondrial swelling, cristae fragmentation, and membrane rupture [45]. In this study, transmission electron microscopy (TEM) was used for high-resolution observation of the ultrastructure of colon tissues. It was found that DSS-induced UC mice showed a significant reduction in the number of mitochondria and an obvious loss of mitochondrial cristae in colonic epithelial cells. In contrast, EA treatment effectively mitigated DSS-induced mitochondrial damage and improved the integrity of the colonic epithelial cell membrane (Fig. 3I). No such changes were observed in the SEA group. In summary, these results indicate that EA treatment not only effectively reduces colonic inflammation but also significantly enhances antioxidant defense capacity in UC mice.

### EA suppresses ferroptosis in colonic epithelial cells of UC mice

**Fig. 4:**
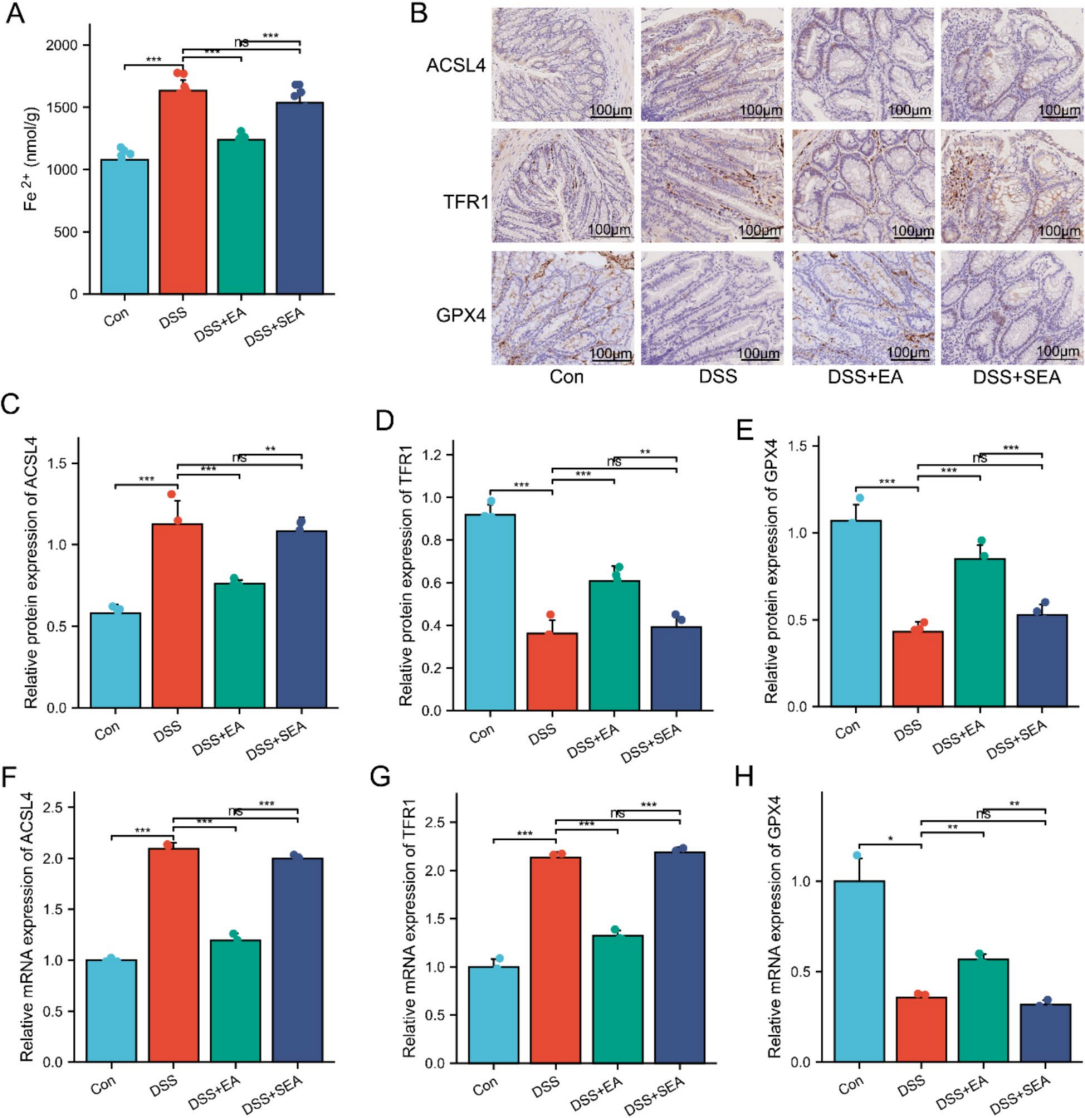
EA suppresses ferroptosis in colonic epithelial cells of UC mice. **A** Fe^2^⁺ levels of Colonic epithelial cells. **B** Immunohistochemical analysis of ACSL4, TFR1, and GPX4 in colonic tissues. Scale bars: 100 μm. **C**–**E** Expression of ACSL4, TFR1, and GPX4 in colonic tissues (n = 4). **F**–**H** mRNA levels of ACSL4, TFR1, and GPX4 in colonic tissues (n = 3). Data are expressed as mean ± SD. ^*ns*^*P* > 0.05, *P < 0.05, **P < 0.01, ***P < 0.001

Ferroptosis, particularly in colonic epithelial cells, has been identified as a potential key regulatory target for UC therapeutic intervention [46]. To investigate whether EA ameliorates symptoms in UC mice by inhibiting ferroptosis in colonic epithelial cells, we further examined the effects of EA on DSS-induced ferroptosis. Results showed a significant increasing trend in colonic epithelial cell Fe^2^⁺ levels in the DSS + EA group compared to the DSS group (Fig. 4A). Immunohistochemistry revealed that the expression of ferroptosis markers TFR1 and ACSL4 was significantly upregulated, while GPX4 expression was significantly downregulated in the DSS and DSS + SEA groups. Notably, EA significantly reversed these aberrant expressions of ferroptosis-related indicators, whereas no significant changes were observed in the SEA group (Fig. 4B–H). These findings indicate that the DSS group significantly exacerbated the ferroptosis process in colonic epithelial cells compared to the Con group, and EA stimulation at ST25 was more effective than SEA in suppressing ferroptosis in these cells.

### Ferroptosis plays a crucial role in the amelioration of EA-mediated UC mice

**Fig. 5:**
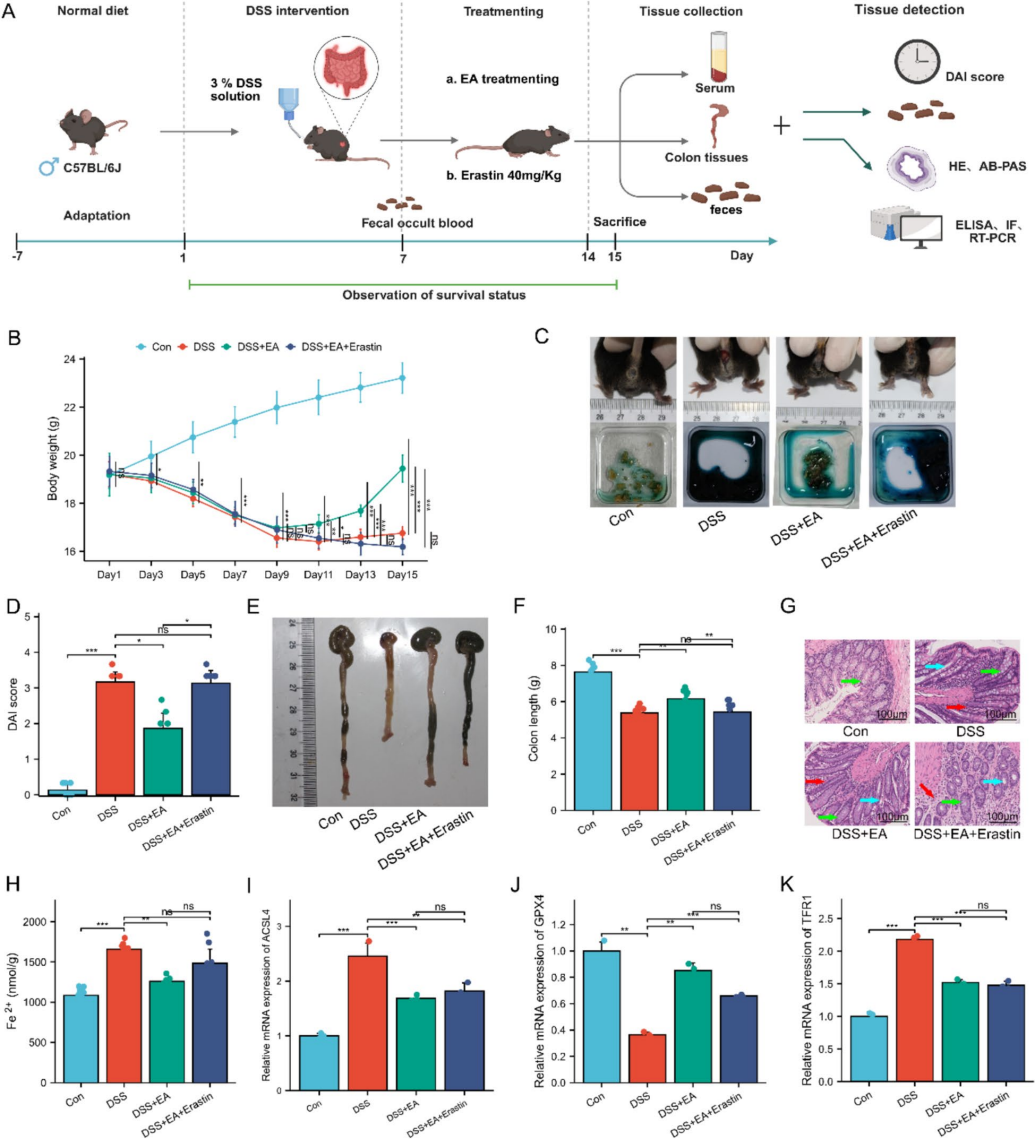
Ferroptosis reversed the colon pathological symptoms of UC mice treated with EA. **A** Experiments protocols. **B** Body weight (n = 10). **C** Appearance of the mouse anus and fecal occult blood test. **D** DAI score (n = 10). **E** Macroscopic view of colons. **F** Colon length (n = 10). **G** HE staining of colonic tissues. Scale bars: 100 μm. Green arrows indicate glandular structures; blue arrows denote crypt structures; red arrows highlight inflammatory cell infiltration. **H** Levels of Fe^2+^ in the colon (n = 10). **I**–**K** mRNA levels of GPX4, ACSL4, and TFR1 in colonic tissues (n = 3). Data are expressed as mean ± SD. ^*ns*^*P* > 0.05, *P < 0.05, **P < 0.01, ***P < 0.001

Based on the observed inhibition of ferroptosis in colonic epithelial cells during EA treatment for UC, we further employed the ferroptosis-specific inducer erastin in a co-treatment strategy to inversely verify the critical regulatory role of ferroptosis in the EA-mediated suppression of UC progression. The specific timeline for the second-phase animal experiment is detailed in Fig. 5A. Experimental results demonstrated that erastin significantly antagonized the protective effects of EA in DSS-induced UC mice. This was specifically manifested as a significant weight loss, increased DAI, markedly shortened colon length, and aggravated histopathological damage in colon tissues (Fig. 5B–G). Notably, erastin almost completely reversed the inhibitory effects of EA on ferroptosis in colonic epithelial cells of UC mice, evidenced by significantly increased Fe^2^⁺ levels in the colon tissue of the DSS + EA + Erastin group (Fig. 5H). As expected, erastin also reversed EA’s regulatory effects on key ferroptosis markers, including GPX4, TFR1, and ACSL4 (Fig. 5I–K).

**Fig. 6:**
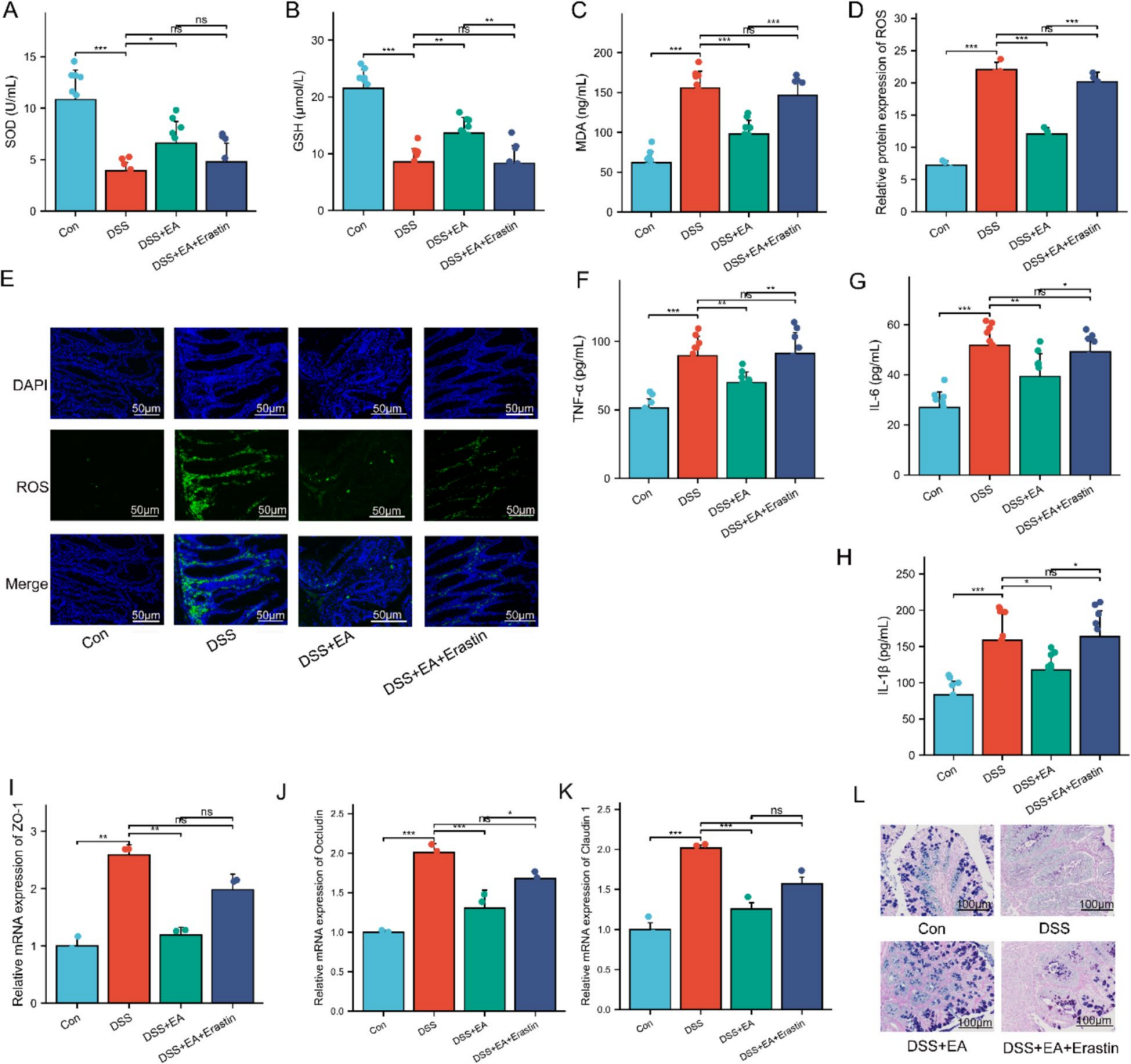
Ferroptosis reversed the colonic barrier function of UC mice treated with EA. **A**–**C** Levels of SOD, GSH, and MDA in the colon (n = 10). **D**, **E** Immunofluorescence analysis of ROS (green) in colonic tissues (n = 4). Cell nuclei were stained with DAPI (blue). Scale bars: 50 μm. **F**, **G** Serum levels of TNF-α and IL-6 (n = 10). **H** AB-PAS staining of colonic tissues. Scale bars: 100 μm. **I** Serum levels of IL-1β (n = 10). **J**–**L** mRNA levels of ZO-1, Occludin, and Claudin 1 in colonic tissues (n = 3). Data are expressed as mean ± SD. ^*ns*^*P* > 0.05, *P < 0.05, **P < 0.01, ***P < 0.001

Furthermore, we assessed changes in oxidative stress markers (SOD, GSH, MDA, and ROS). The results indicated that erastin reversed the antioxidant effects of EA by decreasing serum SOD and GSH levels, increasing MDA content, and concurrently downregulating ROS expression levels in colon tissue (Fig. 6A–E). Given that EA also possesses protective effects, including alleviating colitic inflammation and enhancing intestinal barrier function, this study further explored the impact of erastin on EA’s regulation of the inflammatory response and intestinal barrier integrity. Results showed that erastin significantly reversed the inhibitory effects of EA on pro-inflammatory cytokines (IL-1β, IL-6, and TNF-α) (Fig. 6F–H). Simultaneously, erastin reversed the EA-mediated upregulation of tight junction proteins ZO-1, Occludin, and Claudin-1 (Fig. 6I–K), AB-PAS staining confirmed that erastin counteracted the protective effects of EA on goblet cell numbers and the integrity of the intestinal mucus layer (Fig. 6L), suggesting that erastin significantly undermined the protective effects of EA on intestinal barrier function.

### JAK2/STAT3 signaling pathway participates in EA-regulated ferroptosis in colonic epithelial cells of UC mice

**Fig. 7:**
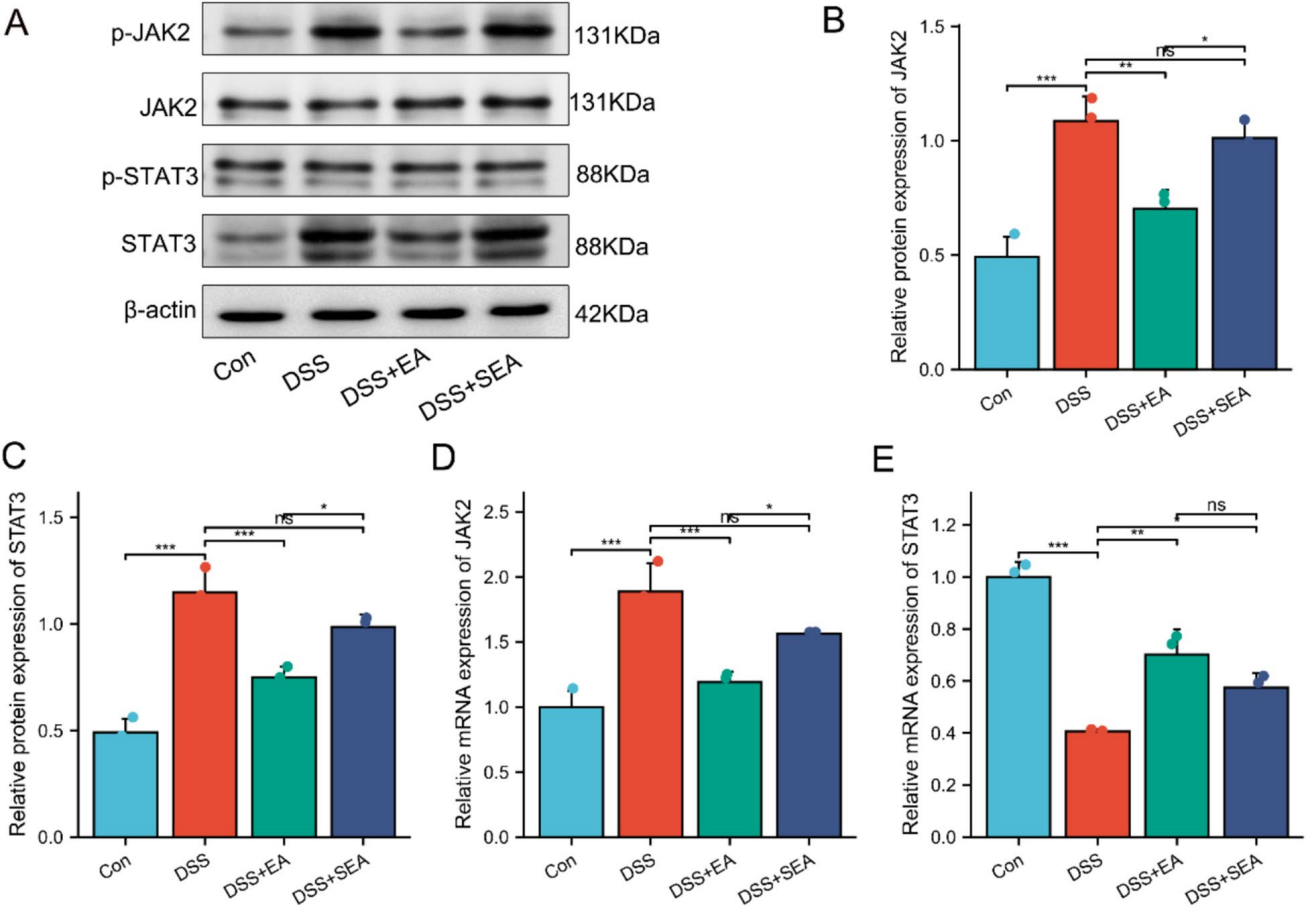
JAK2/STAT3 signaling pathway participates in EA-regulated ferroptosis in colonic epithelial cells of UC mice. **A**–**C** Protein levels of JAK2 and STAT3 in colonic tissues (n = 3). **D**, **E** mRNA levels of JAK2 and STAT3 in colonic tissues (n = 3). Data are expressed as mean ± SD. ^*ns*^*P* > 0.05, *P < 0.05, **P < 0.01, ***P < 0.001

Previous studies have shown that modulating the expression of the JAK2/STAT3 signaling pathway can further regulate the expression of key regulators such as SLC7A11 and GPX4, and influence iron homeostasis via pathways including ferritinophagy and hepcidin regulation, thereby promoting ferroptosis [47]. Therefore, we hypothesized that acupuncture might alleviate UC pathological symptoms by regulating ferroptosis through the JAK2/STAT3 signaling pathway. We detected the protein and mRNA expression levels of JAK2 and STAT3 in colon tissue. WB results indicated that compared to the Con group, the protein expression levels of JAK2 and STAT3 were significantly upregulated in the DSS group. In contrast, these levels were significantly downregulated in the DSS + EA group compared to the DSS group. Notably, the DSS + EA group demonstrated a more pronounced regulatory effect compared to the DSS + SEA group (Fig. 7A–C). RT-PCR analysis further revealed that the mRNA expression levels of JAK2 and STAT3 in the colon tissues of the DSS + EA group also followed a similar trend (Fig. 7D, E). In summary, these results indicate that EA effectively downregulates the expression of JAK2 and STAT3, whereas DSS exerts the opposite effects.

### The JAK2 inhibitor AG490 mimics the therapeutic effects of EA in UC mice

**Fig. 8:**
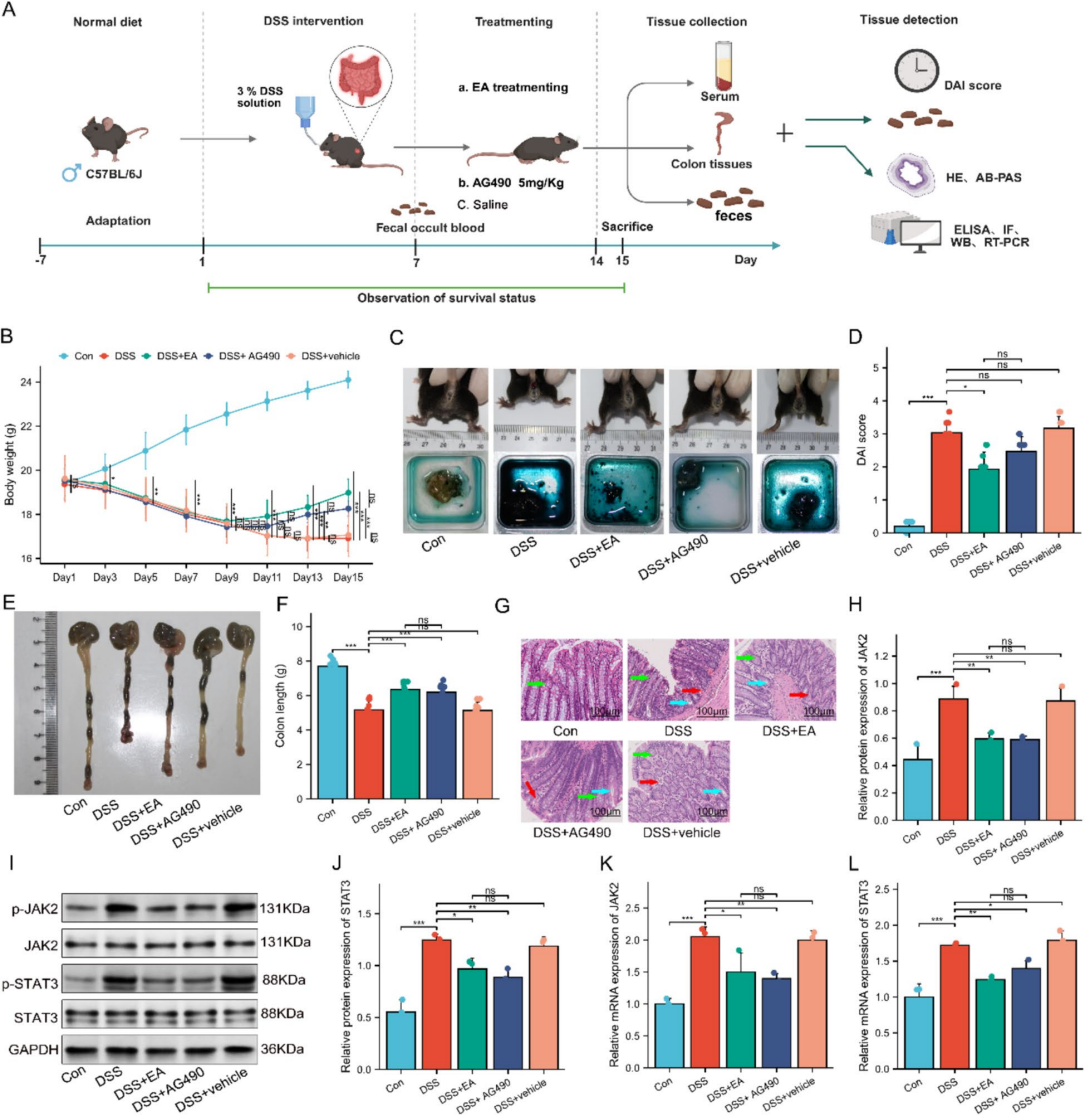
The JAK2 inhibitor AG490 mimics the therapeutic effects of EA on colonic pathology in UC mice. **A** Experiments protocols. **B** Body weight (n = 10). **C** Appearance of the mouse anus and fecal occult blood test. **D** DAI score (n = 10). **E** Macroscopic view of colons. **F** Colon length (n = 10). **G** HE staining of colonic tissues. Scale bars: 100 μm. Green arrows indicate glandular structures; blue arrows denote crypt structures; red arrows highlight inflammatory cell infiltration. **H**–**J** Protein levels of JAK2 and STAT3 in colonic tissues (n = 3). **K**, **L** mRNA levels of JAK2 and STAT3 in colonic tissues (n = 3). Data are expressed as mean ± SD. ^*ns*^*P* > 0.05, *P < 0.05, **P < 0.01, ***P < 0.001

As mentioned, to further verify that acupuncture primarily alleviates the DSS-induced UC phenotype in mice by mediating ferroptosis via the JAK2/STAT3 signaling pathway, we employed the specific JAK2/STAT3 pathway inhibitor AG490 for intervention. The specific timeline for the third-phase animal experiment is detailed in Fig. 8A. In the comparative analysis of therapeutic effects, AG490 was able to mimic the improvement effects of EA treatment on UC pathological features. This was specifically manifested as a significantly decreased DAI, markedly increased colon length, and improved histopathological damage in colon tissues (Fig. 8B–G). In addition, after inhibiting JAK2 and STAT3 with AG490, both the protein and mRNA expression levels of JAK2 and STAT3 in the DSS + AG490 group were significantly downregulated compared to the DSS group. Notably, the DSS + EA group exhibited similar changes in the protein and mRNA expression levels of JAK2 and STAT3, suggesting that EA likely exerts its therapeutic effects by downregulating JAK2 and its downstream target STAT3, and these effects demonstrated comparable efficacy to AG490 intervention (Fig. 8H–J). RT-PCR results further validated that the expression trends of JAK2 and STAT3 were consistent with the WB findings (Fig. 8K, L). In summary, AG490 intervention significantly ameliorated symptoms in DSS-induced UC mice, implying that the JAK2/STAT3 signaling pathway plays an important role in UC development. It is noteworthy that although the DSS + AG490 group showed a trend towards better outcomes than the DSS + EA group, the difference was not statistically significant.

**Fig. 9:**
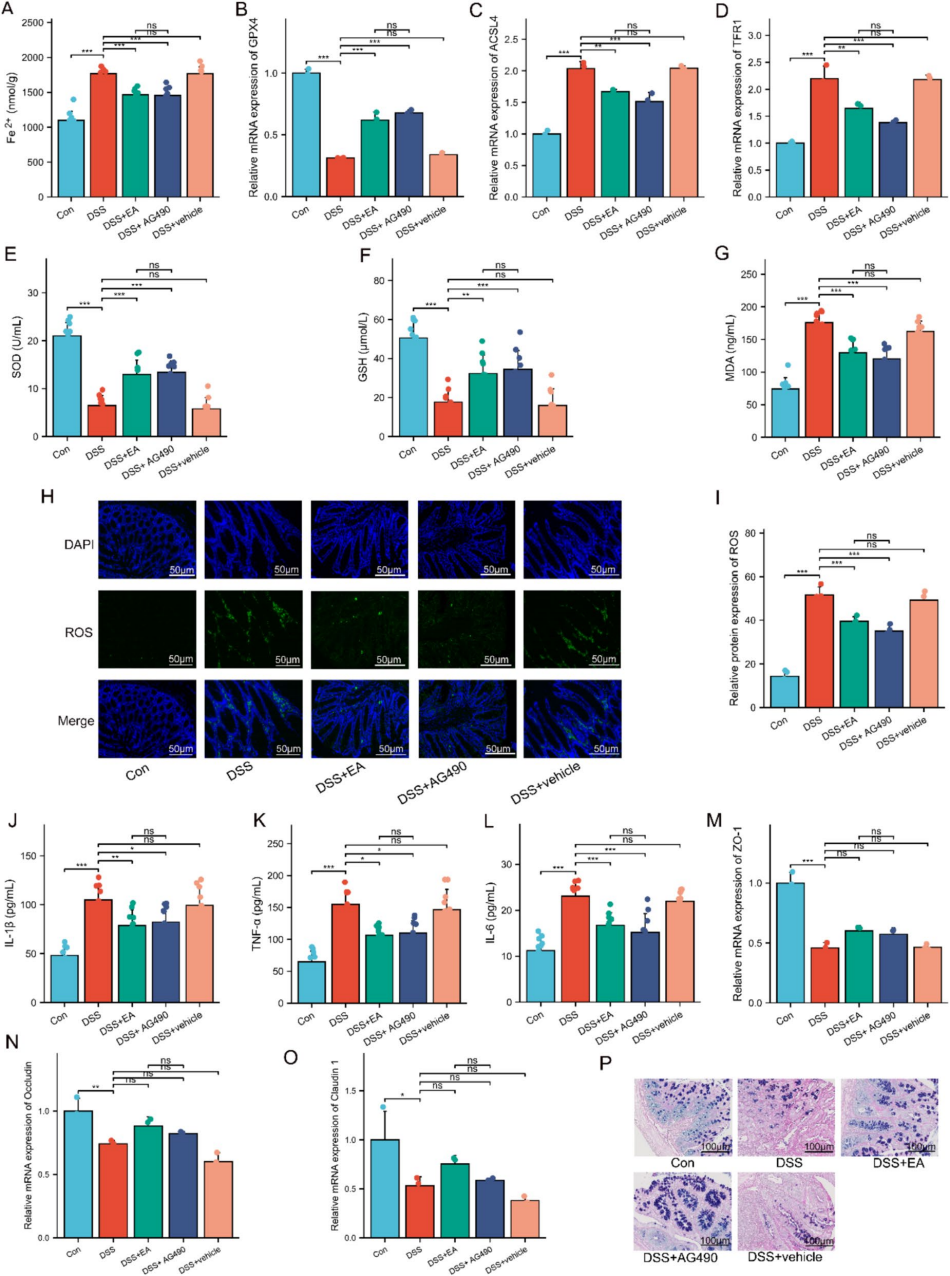
The JAK2 inhibitor AG490 mimics the therapeutic effects of EA on the colonic barrier in UC mice. **A** Levels of Fe^2+^ in the colon (n = 10). **B**–**D** mRNA levels of GPX4, ACSL4, and TFR1 in colonic tissues (n = 3). **E**–**G** Levels of SOD, GSH, and MDA in the colon (n = 10). **H**, **I** Immunofluorescence analysis of ROS (green) in colonic tissues (n = 4). Cell nuclei were stained with DAPI (blue). Scale bars: 50 μm. **J**–**L** Serum levels of TNF-α, IL-6, and IL-1β (n = 10). **M**–**O** mRNA levels of ZO-1, Occludin, and Claudin 1 in colonic tissues (n = 3). **P** AB-PAS staining of colonic tissues. Scale bars: 100 μm. Data are expressed as mean ± SD. ^*ns*^*P* > 0.05, *P < 0.05, **P < 0.01, ***P < 0.001

In addition, we evaluated changes in markers associated with ferroptosis, oxidative stress, inflammatory response, and intestinal barrier function. Specifically, compared to the DSS group, both the DSS + EA and DSS + AG490 groups showed significantly reduced Fe^2^⁺ content in colon tissue (Fig. 9A), accompanied by significantly upregulated expression of ferroptosis-inhibiting genes (GPX4) and significantly downregulated expression of the pro-ferroptosis gene (ACSL4, TFR1) (Fig. 9B–D). Both groups exhibited similar improving trends in the levels of oxidative stress markers (SOD, GSH, MDA, ROS), the expression of inflammatory cytokines (IL-1β, TNF-α, IL-6), and the regulation of intestinal mucosal barrier tight junction protein expression (ZO-1, Occludin, and Claudin-1) (Fig. 9E–O). AB-PAS staining confirmed that AG490 mimicked the protective effects of EA on goblet cell numbers and the integrity of the intestinal mucus layer (Fig. 9P). These findings reveal that regulation of the JAK2/STAT3 signaling pathway is a key mechanism underlying the therapeutic effects of acupuncture in UC.

## Discussion

**Fig. 10:**
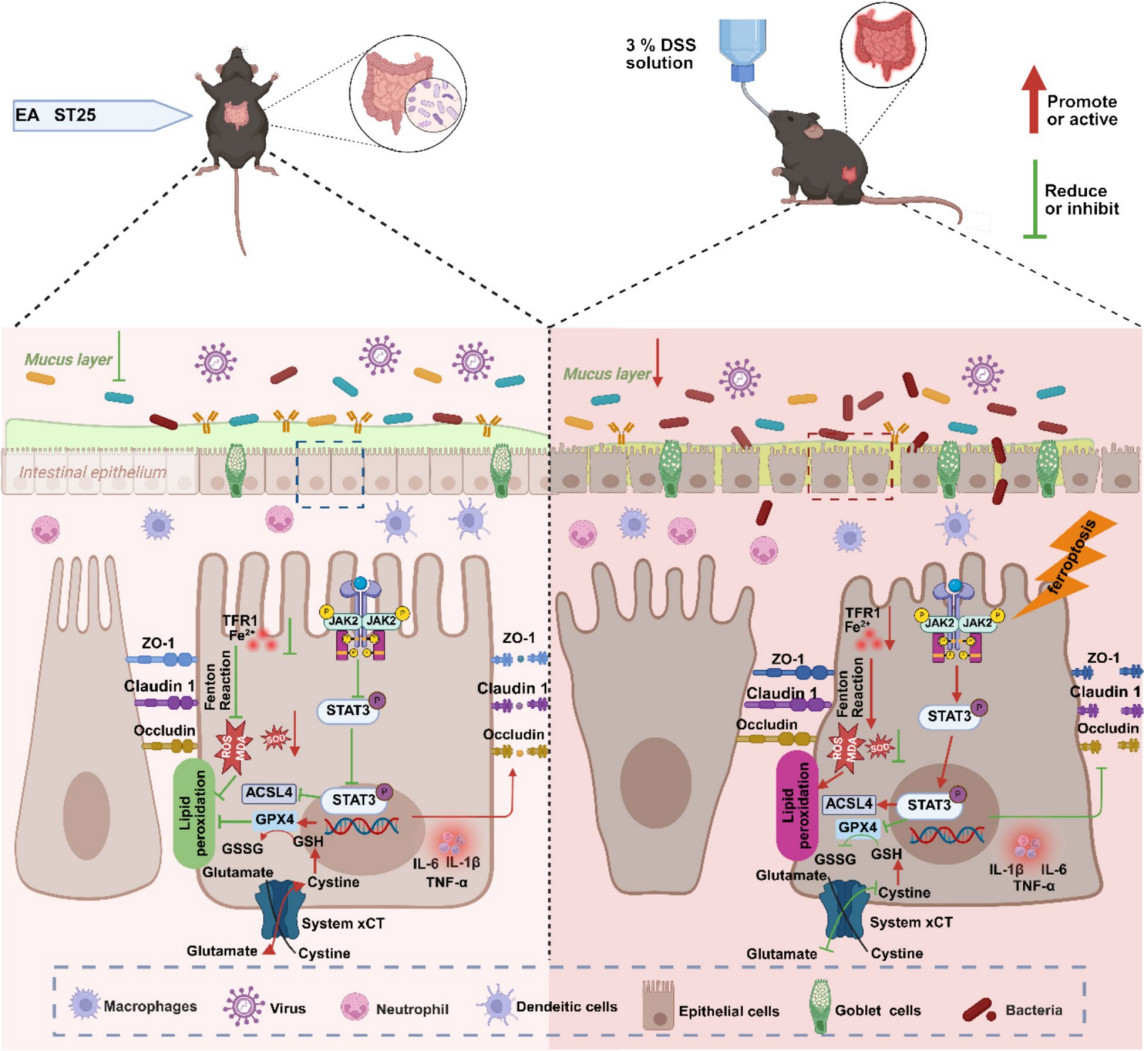
EA alleviates UC symptoms through a mechanism primarily involving the suppression of the JAK2/STAT3 signaling pathway, thereby effectively inhibiting the ferroptosis process. Concurrently, it mitigates colonic tissue damage induced by oxidative stress and suppresses the inflammatory cascade, ultimately exerting protective effects on the colonic mucosa and maintaining the integrity of the intestinal barrier

In this study, we systematically investigated the functional effects of EA in treating UC and its underlying mechanisms (Fig. 10). The main findings are as follows: (1) EA effectively ameliorated intestinal mucosal barrier dysfunction, oxidative stress, inflammatory response, and the ferroptosis process in UC; (2) The ferroptosis activator erastin could significantly reverse the protective effects of acupuncture against UC by exacerbating intestinal mucosal barrier damage, oxidative stress, and the inflammatory cascade; (3) The anti-colitis effects of EA may be mediated through inhibition of the JAK2/STAT3 pathway, suggesting this pathway is one of the potential key mechanisms of its action. In summary, these findings provide novel insights into the clinical diagnosis and treatment of UC and offer a scientific basis for the clinical application of acupuncture in UC management.

Recent studies on DSS-induced UC models have reported a marked and progressive body weight loss in mice following DSS administration [48]. Our experimental results further validate this phenomenon. Notably, rapid intervention with EA after model establishment not only significantly attenuated the degree of weight loss but also effectively improved the pathological manifestation of colon shortening. These findings suggest that EA stimulation at ST25 may alleviate DSS-induced intestinal damage through a protective mechanism. However, mice in the DSS + SEA group did not exhibit significant recovery trends in either body weight or colon length metrics, which may be related to the biological specificity of acupoint selection. The precise underlying mechanisms remain unclear and warrant further in-depth investigation.

The impairment of the intestinal mucosal barrier, a hallmark pathological feature of UC, is a critical event in its onset and progression, with its integrity encompassing intestinal epithelial cells, tight junctions, the mucus layer, and immune cells [49, 50]. Compared to previous studies, this study conducted a more comprehensive evaluation of intestinal barrier integrity: H&E staining revealed inflammatory cell infiltration, confirming that EA effectively alleviated damage to intestinal epithelial cells; immunohistochemical detection further demonstrated that EA upregulates the expression levels of tight junction proteins; and AB-PAS staining clearly confirmed a significant protective effect of EA on the mucus layer. These findings indicate that acupuncture inhibits the DSS-induced increase in intestinal barrier permeability, thereby exerting a protective effect on the intestinal mucosal barrier.

Oxidative stress and inflammatory response are major pathogenic factors in UC [51, 52]. Notably, oxidative stress can also exacerbate colonic histopathological damage in UC by promoting the aberrant release of pro-inflammatory cytokines such as IL-1β, IL-6, and TNF-α [53]. Given this connection, inflammatory factor assays further indicated that acupuncture intervention effectively suppressed the colonic inflammatory response in UC mice. However, given the multi-target regulatory nature of acupuncture and the multifaceted pathogenesis of UC, single-dimensional mechanistic approaches are inadequate to fully elucidate the therapeutic efficacy of acupuncture in UC or its potential clinical relevance. Therefore, it is imperative to establish a core-oriented integrated hypothesis to elucidate the molecular mechanism underlying acupuncture’s inhibition of UC progression, systematically integrating key pathological aspects, including intestinal barrier dysfunction, oxidative stress, inflammatory cascades, and immune regulation, to form a coherent explanatory framework.

Within the ferroptosis regulatory network, GPX4, a classical negative regulator, primarily exerts its protective role by maintaining intracellular GSH levels [54]. Under conditions of GPX4 deficiency, cellular iron homeostasis becomes more susceptible to disruption [54]. The resultant iron accumulation exacerbates the Fenton reaction and promotes lipid peroxidation, thereby driving the process of ferroptosis [55]. ACSL4, a key marker of redox status, can reflect the level of oxidative stress driving ferroptosis [56]. Furthermore, intracellular iron ions are a necessary condition for ferroptosis, and their content increases significantly during the process [57]. Excess Fe^2^⁺ plays a crucial role in the occurrence of ferroptosis by interacting with ROS generated from lipid peroxidation [57]. Related studies also indicate that changes in intracellular Fe^2^⁺ concentration, alterations in mitochondrial morphology, and dynamic changes in oxidative stress biomarkers can serve as important detection indicators for ferroptosis [14]. Notably, the combined application of the ferroptosis agonist erastin with EA intervention almost completely abolished the protective effects of acupuncture on intestinal mucosal barrier dysfunction, oxidative stress, and the inflammatory cascade in UC mice. More crucially, this study reveals for the first time that ferroptosis serves as a pivotal regulatory mechanism and core molecular pathway through which acupuncture exerts its protective effects on the colonic barrier, as well as its antioxidant and anti-inflammatory activities. This key finding is consistent with recent literature, which has demonstrated that acupuncture similarly suppresses ferroptosis in other inflammatory disease models, such as arthritis and sepsis, further corroborating the converging evidence in this field.

Ferroptosis, oxidative stress, and inflammatory responses are closely interconnected, forming a complex regulatory network [58]. Dysregulation of these mechanisms may collectively contribute to the pathogenesis of UC. Our study demonstrates that the primary molecular mechanism through which acupuncture alleviates UC involves the suppression of the JAK2/STAT3 signaling pathway. As transcription factors with multiple functions, JAK2 and STAT3 play pivotal regulatory roles in inflammatory responses [59], oxidative stress [60], and the ferroptosis process [47], making them significant targets in UC pathogenesis. Given the comprehensive regulatory function of the JAK2/STAT3 pathway and the multi-target nature of acupuncture, a potential connection between acupuncture and this signaling pathway appears plausible. Although existing reports on the relationship between the JAK2/STAT3 pathway and ferroptosis regulation remain somewhat controversial, JAK2, STAT3 phosphorylation may either promote or inhibit ferroptosis progression depending on the specific disease model and physiological state of the microenvironment. For instance, in a mouse model of postoperative ileus (POI), acupuncture was reported to activate the JAK2/STAT3 pathway, upregulate p-JAK2 and p-STAT3 expression, inhibit GABA receptor expression in DMV neurons, and subsequently suppress inflammation induced by intestinal manipulation [61]. Furthermore, a recent study on colitis found that DSS-induced intestinal epithelial injury significantly activated ferroptosis, whereas a ferroptosis inhibitor alleviated colonic epithelial cell damage by upregulating p-JAK2 and p-STAT3 [62]. While our findings indicate that acupuncture inhibits the aberrant activation of the JAK2/STAT3 pathway, thereby significantly ameliorating ferroptosis, intestinal mucosal barrier dysfunction, oxidative stress, and local colonic inflammation. These results suggest that targeted inhibition of the JAK2/STAT3 pathway is a key mechanism regulating ferroptosis in colonic epithelial cells. Related literature confirms that berberine ameliorates murine colitis by regulating ferroptosis via the JAK2 signaling pathway, validated using the JAK2 inhibitor AG490 [63]. In our study, we further employed AG490 to intervene in the JAK2/STAT3 pathway, confirming that this pathway is a key mechanism in acupuncture treatment for UC. In summary, these findings provide compelling evidence elucidating the biological effects and molecular mechanisms of acupuncture in treating UC.

This study used the tail as the non-acupoint control site. The primary advantages of this choice are threefold. Firstly, it effectively avoids all known meridians, ensuring a theoretical distinction from acupoint stimulation. Secondly, its location outside the torso facilitates EA application without interfering with the acupoint group and allows for easy immobilization. Finally, this approach is widely adopted, facilitating comparison of our results with prior literature [35, 38]. However, we acknowledge the potential limitations of this strategy. The local anatomy of the tail (e.g., skin thickness, subcutaneous tissue, nerve distribution) differs from that of the acupoint site (e.g., ST25 on the abdomen), which may evoke different stimulation sensations [64]. Although identical EA parameters (intensity, frequency, duration) were maintained to control variables, the influence of these anatomical differences on the results cannot be entirely ruled out. It is also important to note the ongoing debate surrounding the concept of a “non-acupoint.” Some perspectives argue that any stimulation of the body surface may produce physiological effects via broad somatic sensory afferents, making a truly “neutral” control difficult to achieve. Thus, tail stimulation might be considered a “weak” or “heterotopic” stimulus rather than a completely inert control. Other common non-acupoint choices in research (e.g., non-meridian points or non-specific points adjacent to acupoints) may better match local anatomy but risk inadvertently stimulating neighboring meridians. Therefore, our selection represents a balance between traditional Chinese medical theory and practical feasibility, and it robustly demonstrates that the effects of ST25 stimulation are relatively specific.

In this study, EA may alleviate ferroptosis in colonic epithelial cells by inhibiting the JAK2/STAT3 pathway, significantly reducing oxidative stress injury, improving intestinal mucosal barrier integrity, and inhibiting the DSS-induced inflammatory cascade in UC mice. However, this study has several limitations. Firstly, the investigation into acupuncture’s role in inhibiting cellular ferroptosis is constrained by the absence of a ferroptosis inhibitor as a positive control. This lack of comparative control may affect the rigor of result interpretation, as direct comparison with established ferroptosis inhibitors (such as ferrostatin-1 or liproxstatin-1) could provide more compelling evidence to distinguish the specific anti-ferroptosis effects of acupuncture from general cytoprotective mechanisms. Subsequent studies should incorporate pharmacological ferroptosis inhibitors to validate the conclusion that acupuncture suppresses iron-dependent programmed cell death through targeted molecular pathway verification. Secondly, to validate the molecular mechanism of the JAK2/STAT3 signaling pathway, we employed only the JAK2 inhibitor AG490 for intervention without including a DSS + EA + AG490 as a control. This might render the mechanistic validation dimensionally insufficient. Finally, this study primarily focused on ferroptosis and has not yet addressed other cell death modalities, such as pyroptosis and apoptosis, or regulatory networks like the immune microenvironment, thus failing to comprehensively elucidate the integrated therapeutic mechanisms of electroacupuncture. Future studies need to introduce rescue experiments using pathway activators (e.g., IL-6).

## Conclusion

In summary, our research suggests that acupuncture may alleviate the symptoms of UC by inhibiting the JAK2/STAT3 signaling pathway, thereby effectively suppressing the process of ferroptosis. Concurrently, it mitigates colonic tissue damage induced by oxidative stress and suppresses the inflammatory cascade, ultimately exerting protective effects on the colonic mucosa and maintaining the integrity of the intestinal barrier. These findings indicate that acupuncture, as a safe and effective external therapy, holds promising potential for clinical application in the treatment of UC.

## Supplementary Information

Supplementary material 1.

## Data Availability

The data associated with this study can be obtained from the corresponding author upon reasonable request.

## Abbreviations

UC: Ulcerative colitis
EA: Electroacupuncture
DAI: Disease activity index
IHC: Immunohistochemistry
IF: Immunofluorescence
WB: Western blotting
HE: Hematoxylin–eosin
IL‑1β: Interleukin‑1β
IL‑6: Interleukin‑6
TNF-α: Tumor necrosis factor-α
GSH: Glutathione
SOD: Superoxide dismutase
MDA: Malondialdehyde
ROS: Reactive oxygen species
GPX4: Glutathione peroxidase 4
ACSL4: Acyl-CoA synthetase long-chain family member 4
TFR1: Transferrin receptor 1
JAK2: Janus kinase 2
STAT3: Signal transducer and activator of transcription 3
