# Supplementary material for: Electroacupuncture ameliorates ulcerative colitis by suppressing ferroptosis via the JAK2/STAT3 signaling pathway
Source: Chin Med. 2026 Jan 28;21:56. doi: 10.1186/s13020-026-01337-9 (PMC12849387; doi:10.1186/s13020-026-01337-9)
Supplement: Supplementary file 1 — Supplementary material 1. [file 13020_2026_1337_MOESM1_ESM.docx]

Supplementary Table 1 Sores of disease activity index (DAI)

| **Feature** | **Score** | **Description** |
| --- | --- | --- |
| Body weight loss | 0 | 0% |
|  | 1 | 1-5% |
|  | 2 | 5-10% |
|  | 3 | 10-15% |
|  | 4 | >15% |
| Feces status | 0 | Normal |
|  | 2 | Loose stools |
|  | 4 | Watery stool |
| Occult/Bloody stools | 0 | Normal |
|  | 2 | Hemoccult positive |
|  | 4 | Hematochezia with nakedeyes |

Supplementary Table 2 Immunohistochemical (IHC) and Immunofluorescence (IF)

| Producer | Name | Article number | Dilution ratio |
| --- | --- | --- | --- |
| Proteintech | ZO-1 Polyclonal antibody | 21773-1-AP | 1：1000 |
|  | Occludin Polyclonal antibody | 27260-1-AP | 1：2000 |
|  | Claudin 1 Polyclonal antibody | 28674-1-AP | 1：1000 |
|  | CD71 Polyclonal antibody (TFR1) | 32333-1-AP | 1：1000 |
|  | GPX4 Monoclonal antibody | 67763-1-Ig | 1：2000 |
|  | ACSL4/FACL4 Polyclonal antibody | 22401-1-AP | 1：200 |
|  | Anti-PRDM1/Blimp1 antibody [ROS195G] | ab241568 | 1：500 |

Supplementary Table 3 Antibody information of Western Blot (WB).

| Producer | Name | Article number | Dilution ratio |
| --- | --- | --- | --- |
| Cell Signaling Technology | Jak2 (D2E12) XP^®^ Rabbit mAb | 3230 | 1：1000 |
|  | Phospho-Jak2 (Tyr1007/1008) Antibody | 3771 | 1：2000 |
|  | Stat3 (124H6) Mouse mAb | 9139 | 1：1000 |
|  | Phospho-Stat3 (Tyr705) (D3A7) XP® Rabbit mAb | 9145 | 1：2000 |
| Proteintech | Beta Actin Monoclonal antibody | 66009-1-Ig | 1：20000 |
|  | GAPDH Monoclonal antibody | 60004-1-Ig | 1：50000 |

Supplementary Table 4 Real-time Quantitative Polymerase Chain Reaction (RT-PCR) primer name and sequence.

| Primer name | Sequence (5' -> 3') |
| --- | --- |
| *JAK2-F* | GCGACGGGAACAAGATGTGA |
| *JAK2-R* | TTCAGAACATCGGCCTTCCC |
| *STAT3-F* | ACCATTGACCTGCCGATGT |
| *STAT3-R* | GATCCATGTCAAACGTGAGCG |
| *ZO-1-F* | ACAGCCAGCTCTTGGTCATC |
| *ZO-1-R* | GTATGGTGGCTGCTCAAGGT |
| *Claudin 1-F* | CTGTCCCCGGAAAACAACCT |
| *Claudin 1-R* | GCACAGCCAAGACCCTCATA |
| *Occludin-F* | TAACCCACTAGACCTTTCCATTGT |
| *Occludin-R* | CAGAACCCAAGACAGGTCACA |
| *ACSL4-F* | GCGCTCCTCTTATTTGCTGTG |
| *ACSL4-R* | AGACACGTACTCTCCGGCT |
| *GPX4-F* | CCGTCTGAGCCGCTTATTGAA |
| *GPX4-R* | CGGTTTTGCCTCATTGCGAG |
| *TFR1-F* | GTTTCTGCCAGCCCCCTATT |
| *TFR1-R* | CACCTCTGCTGCTGTACGAA |
| *GAPDH-F* | ACTCTACCCACGGCAAGTTC |
| *GAPDH-R* | TGGGTTTCCCGTTGATGACC |
